# Neutrophil-to-lymphocyte ratio as a biomarker for asthma identification and severity stratification: a systematic review and meta-analysis

**DOI:** 10.3389/fmed.2025.1620695

**Published:** 2025-06-24

**Authors:** Lei Jin, Jie Guo, Keting Deng, Yang Yao

**Affiliations:** ^1^Department of Cardiothoracic Surgery, The First Affiliated Hospital of Xi'an Medical University, Xi’an, Shaanxi, China; ^2^Department of Emergency, The First Affiliated Hospital of Xi'an Medical University, Xi’an, Shaanxi, China; ^3^Department of Laboratory Medicine, The First Affiliated Hospital of Xi'an Medical University, Xi’an, Shaanxi, China; ^4^Department of Respiratory and Critical Care Medicine, The First Affiliated Hospital of Xi'an Medical University, Xi’an, Shaanxi, China

**Keywords:** asthma, neutrophil-to-lymphocyte ratio, severity stratification, systemic inflammation, meta-analysis

## Abstract

**Background:**

Reliable biomarkers for asthma identification and severity stratification remain lacking. The neutrophil-to-lymphocyte ratio (NLR) has emerged as a potential candidate, but evidence remains inconsistent. This study evaluates the value of NLR in distinguishing asthma patients from healthy controls and its correlation with disease severity.

**Methods:**

A systematically search was conducted across PubMed, Embase and Web of Science for studies reporting NLR levels in asthma patients and healthy controls. Pooled mean differences (MD) and 95% confidence intervals (CIs) were calculated using random-effects models. Receiver operating characteristic (ROC) curves assessed discriminative performance.

**Results:**

Nineteen studies (43,164 patients, 8,411 controls) were included. When comparing across different asthma severities, the NLR showed incremental increases across severity: mild vs. moderate asthma (MD = −0.41, 95% CI: −0.64 to −0.18, *p* = 0.0005), mild vs. severe (MD = −3.10, 95% CI: −6.26 to 0.06, *p* = 0.05), and moderate asthma vs. severe asthma (MD = −2.44, 95% CI: −5.31 to 0.44, *p* = 0.10). The comparison between severe and non-severe asthma also showed a significant difference (MD = −2.06, *p* < 0.0001). NLR robustly discriminated asthma from controls (AUC = 0.929) and severe from non-severe asthma (AUC = 0.914). Subgroup analyses revealed higher NLR differences in pediatric populations and developed regions.

**Conclusion:**

NLR is a promising biomarker for asthma and severity stratification, although its discriminative ability between moderate and severe stages is limited. Future studies should explore its role in predicting asthma progression and exacerbations.

## Introduction

Asthma is a chronic respiratory disease characterized by airway inflammation and reversible airflow obstruction (1), affects over 300 million individuals worldwide and imposes a substantial burden on healthcare systems. Asthma is typically managed with a stepwise approach centered on anti-inflammatory therapy, especially inhaled corticosteroids (ICS) (2), For patients with more severe disease, additional treatments such as long-acting *β*₂-agonists (LABAs), leukotriene receptor antagonists (LTRAs), or biologic agents may be used (3). However, therapeutic optimization depends on timely and accurate diagnosis, which remains a challenge, highlighting the need for reliable biomarkers. Traditional diagnostic methods, such as spirometry, are useful for assessing airflow limitation but are not effective for early detection or predicting the occurrence of asthma, particularly when symptoms are not yet evident (4), underscoring the urgent need for accessible, cost-effective biomarkers to guide clinical decision-making.

The neutrophil-to-lymphocyte ratio (NLR), a simple and cost-effective marker of systemic inflammation, has emerged as a candidate biomarker for asthma (5). NLR reflects the interplay between neutrophilic and lymphocytic immune pathways-key drivers of asthma heterogeneity (6). While preliminary studies suggest elevated NLR correlates with asthma severity and exacerbations (7, 8), existing evidence is limited by methodological inconsistencies, insufficient exploration of confounding factors (e.g., age, geographic disparities), and a lack of standardized thresholds for clinical application.

This systematic review and meta-analysis aims to compare NLR levels between asthma patients and healthy controls, and to examine its variation across subgroups stratified by asthma severity. Additionally, we will investigate how demographic factors such as age and geographic region influence NLR differences. By synthesizing existing evidence, our findings aim to bridge critical knowledge gaps, offering insights for integrating NLR into standardized diagnostic frameworks to enhance precision in asthma management.

## Methods

### Search strategy and selection criteria

A comprehensive literature search was conducted in PubMed, Embase, and Web of Science from inception to 2025 using the following terms: “NLR” OR “neutrophil-to-lymphocyte ratio” OR “neutrophil lymphocyte ratio” OR “neutrophil/lymphocyte ratio” OR “inflammatory biomarker*” AND “asthma” OR “bronchial asthma” OR “asthma exacerbation*. Studies were included if they: (1) Involved asthma patients of any severity, with or without exacerbations, as well as healthy control groups. (2) Reported NLR levels in asthma patients and controls, or among asthma severity subgroups. Studies were excluded if they (1) Did not provide original NLR data or based on non-human subjects. (2) Lacked a clear or confirmed asthma diagnosis or did not include a control group or relevant comparisons between different asthma severity levels.(3) Provided incomplete data such as missing NLR values or sample sizes.

### Data extraction and quality assessment

Two independent reviewers screened the studies and extracted the following data: first author, publication year, country, sample size, participant age, and reported NLR levels. Disagreements were resolved by a third reviewer. The extracted date included first authors’ name, publication year, country, sample size, age, NLR level. Newcastle–Ottawa Scale (NOS) were used to assess the quality of each study, which assesses three aspects: selection (4 items), comparability (1 item), and outcome (3 items). Studies were assigned a score between 0 and 9 stars, with studies scoring 7 or more considered high quality. Studies with 7 or more stars were also regarded as high quality (9).

### Statistical analysis

Meta-analyses were performed to compare NLR levels between asthma patients and healthy controls, as well as among asthma subgroups (non-severe vs. severe; mild, moderate, and severe). Continuous data were expressed as mean differences (MD) with corresponding 95% confidence intervals (CIs), and results were presented in forest plots. Heterogeneity was assessed using the Q statistic (*p* < 0.05) and I^2^ statistics. An I^2^ value < 50% indicated low heterogeneity, allowing the use of a fixed-effects model, otherwise, a random-effects model was used. Sensitivity analyses were performed by sequentially removing each study to assess the robustness of the results. Subgroup analyses were conducted based on participant age (<18 vs. ≥18 years) and country development status (developed vs. developing). Publication bias was evaluate using Begg’s and Egger’s tests and visually through funnel plots. A *p* < 0.05 was considered indicative of significant bias. All statistical analyses were performed using Stata software (version 12.0; StataCorp, Texas, United States).

## Result

**Figure 1:**
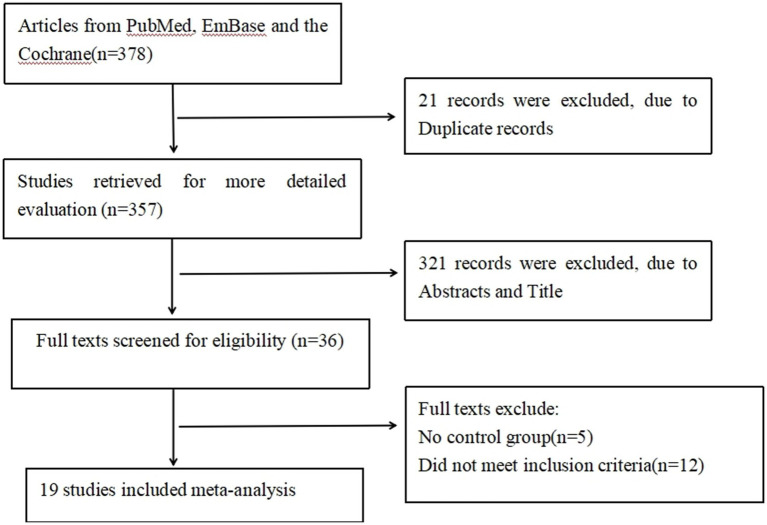
PRISMA flow diagram.

A systematic literature search was conducted in PubMed, EmBase, and the Cochrane Library following PRISMA guidelines. The initial search identified 378 relevant publications. After duplicate removal (*n* = 21), 357 articles were further evaluated based on title and abstract screening, 321 articles were excluded. Full texts evaluation was conducted for remaining 36 articles, with 5 excluded due to the lack of a control group and 12 excluded for not meeting inclusion criteria. Finally, 19 studies were included in the meta-analysis (Figure 1).

### Study characteristics

The baseline characteristic of the included studies and participants are summarized in Tables 1–3. As shown in Table 1, most studies reported significantly higher NLR values in asthma patients compared to healthy controls (5, 6, 10–24). The mean NLR values in the asthma group ranged approximately from 1.80 to 8.58, while in the control group they ranged from 1.01 to 2.42. Table 2 presents the comparison between severe asthma (SA) and non-severe asthma (NSA) groups (25, 26). Across studies, the SA group consistently showed higher NLR values, with reported means ranging from 1.90 to 9.69 in SA, compared to 0.90 to 2.40 in NSA. Table 3 summarizes the NLR across asthma subgroups with varying levels of exacerbation severity (mild, moderate, and severe). An overall increasing trend in NLR values is observed with escalating disease severity. In the majority of included studies, patients with severe asthma consistently exhibit higher mean NLR levels compared to those with moderate or mild forms.

**Table 1:** Demographic characteristics and NLR values in asthma patients and healthy controls.

Author	Year	Country	Sample		Age		NLR	
			Asthma	Control	Asthma	Control	Asthma	Control
Nacaroglu HT (10)	2016	Turkey	54	94	10.00 ± 3.00	14.08 ± 3.33	4.90 ± 8.10	1.50 ± 1.2
Pan RL (11)	2023	China	89	53	6.00 ± 2.96	8.00 ± 2.22	2.97 ± 2.54	1.08 ± 0.54
Dogru (12)	2015	Turkey	469	170	8.58 ± 3.25	8.71 ± 3.03	8.58 ± 3.25	1.77 ± 1.71
Shi G (13)	2017	China	175	130	49.00 ± 14.00	49.51 ± 13.58	1.99 ± 1.15	1.68 ± 0.15
Hendy (14)	2018	Egypt	45	45	37.82 ± 14.54	33.07 ± 10.89	2.77 ± 1.80	1.40 ± 0.52
Zhu XM (6)	2021	China	86	38	6.00 ± 2.96	7.00 ± 2.96	3.08 ± 2.67	1.01 ± 0.46
Beyhan Sagmen S (15)	2019	Turkey	80	22	41.50 ± 11.60	42.00 ± 10.50	1.80 ± 0.67	1.80 ± 0.37
Ke J (5)	2023	China	6,414	41,891	45.36 ± 0.29	47.58 + 0.19	2.00 ± 0.82	2.00 ± 0.82
Wawryk-Gawda E (16)	2023	Poland	375	107	3.72 ± 2.67	6.10 ± 4.17	3.42 ± 4.05	1.94 ± 1.91
Darwesh M (17)	2020	Iraq	50	50	NA	NA	2.06 ± 1.31	2.42 ± 0.50
Singh B (18)	2023	India	50	50	63.00 ± 8.20	62.00 ± 7.90	1.96 ± 0.08	1.08 ± 0.07
Yildiz E (19)	2022	Turkey	150	150	NA	NA	2.80 ± 1.40	1.70 ± 0.50
Tahseen R (20)	2022	India	60	32	41.05 ± 10.38	38.53 ± 6.23	2.37 ± 1.11	1.53 ± 0.54
Gungen AC (21)	2017	Turkey	142	104	48.40 ± 11.40	51.80 ± 13.10	2.20 ± 1.20	1.83 ± 1.02
Sobeih A (22)	2024	Egypt	44	44	10.89 ± 3.57	10.31 ± 2.53	2.23 ± 0.48	1.64 ± 0.38
Bedolla-Barajas M (23)	2022	Mexico	53	109	33.8 ± 12.0	32.4 ± 10.4	1.80 ± 0.61	1.71 ± 0.54
Obeagu EI (24)	2023	Uganda	75	75	NA	NA	4.07 ± 1.06	1.93 ± 0.61

**Table 2:** Demographic and NLR data in severe versus non-severe asthma.

Author	Year	Country	Sample		NLR	
			SA	NSA	SA	NSA
Nacaroglu HT (10)	2016	Turkey	54	54	4.90 ± 8.10	2.40 ± 0.30
Pan RL (11)	2023	China	81	8	4.90 ± 8.10	1.97 ± 4.85
Shi G (13)	2017	China	97	175	9.69 ± 9.84	1.99 ± 1.15
Hendy (14)	2018	Egypt	20	25	3.31 ± 1.97	2.09 ± 1.53
Beyhan Sagmen S (15)	2019	Turkey	46	34	1.90 ± 0.81	1.60 ± 0.44
Darwesh M (17)	2020	Iraq	NM	NM	3.70 ± 1.40	2.20 ± 1.10
Mochimaru T (25)	2018	Japan	46	58	2.48 ± 0.14	2.07 ± 0.13
Asseri AA (26)	2024	Saudi Arabia	128	131	7.00 ± 6.80	0.90 ± 0.70
Gungen (21)	2017	Turkey	69	73	2.51 ± 1.33	2.00 ± 1.10

**Table 3:** Demographic and NLR data among different exacerbated asthma groups.

Author	Year	Country	Sample			NLR		
			Mild	Moderate	Severe	Mild	Moderate	Severe
Pan RL (11)	2023	China	54	17	10	2.67 ± 1.80	3.41 ± 2.67	7.35 ± 6.41
Zhu XM (6)	2021	China	54	17	15	2.74 ± 1.87	3.40 ± 2.47	7.28 ± 3.23
Tahseen R (20)	2022	India	30	30	NA	2.06 ± 0.51	2.68 ± 1.44	NA
Darwesh M (17)	2020	Iraq	NA	NA	NA	0.80 ± 0.70	2.20 ± 1.10	3.70 ± 1.40
Sobeih A (22)	2024	Egypt	24	14	6	1.83 ± 0.39	2.17 ± 0.41	2.59 ± 0.60

### NLR in asthma patients compared to healthy controls

**Figure 2:**
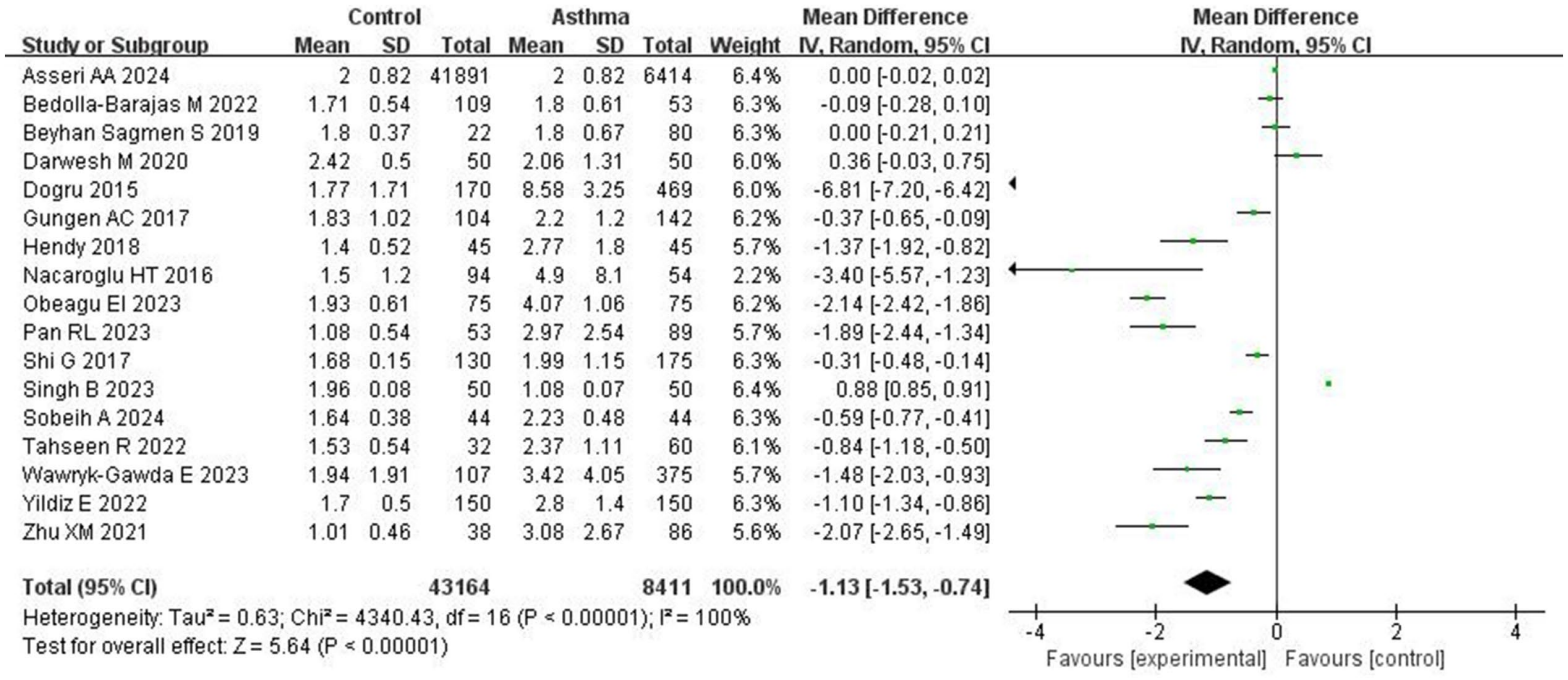
Forest plot comparing the NLR between asthma patients and healthy controls across 17 studies. The mean difference (MD) and 95% confidence intervals (CI) were calculated using a random-effects model.

Seventeen studies investigating the NLR in asthma patients and healthy controls, including a total of 43,164 asthma patients and 8,411 healthy controls, are summarized in Table 2. The reshults showed that asthma patients had significantly higher NLR levels compared control (MD = −1.13; 95% CI: −1.53 to −0.74, *p* < 0.0001), However, there was significant heterogeneity across included studies (I^2^ = 100%; *p <* 0.00001; Figure 2).

### NLR in asthma patients by disease severity

**Figure 3:**
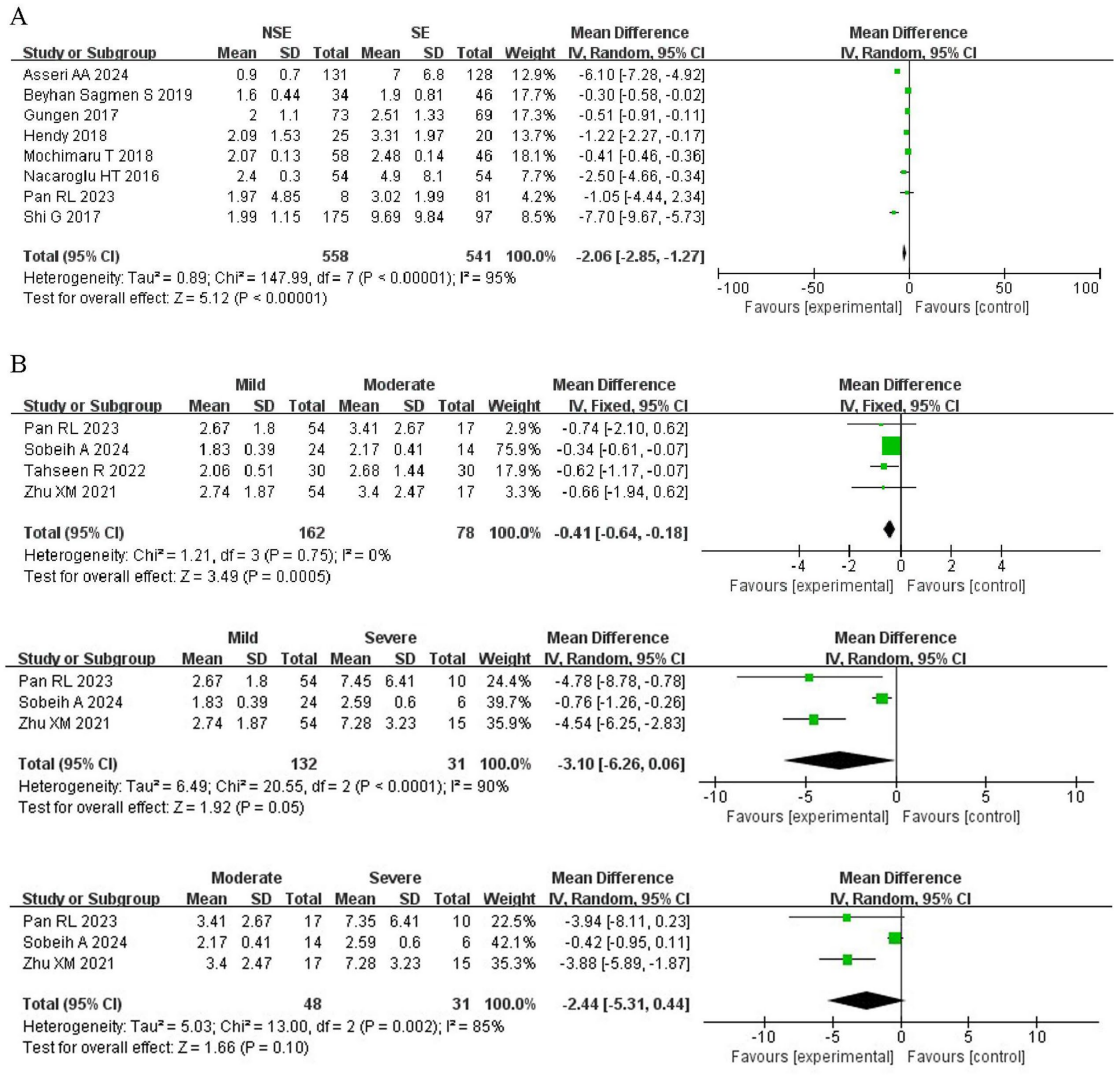
Forest plots of NLR across asthma severity subgroups. **(A)** Comparison between non-severe asthma (NSA) and severe asthma (SA). **(B)** Comparisons among mild, moderate, and severe asthma groups.

The details of the nine studies comparing the NLR between severe asthma (SA) and non-severe asthma (NSA) are summarized in Table 3. These studies involved 558 NSA patients and 541 SE patients. The results showed that patients with SA has significantly higher NLR levels than those with NSA (MD = −2.06; 95% CI: −2.85 to −1.27, *p* < 0.00001; Figure 3A).

In addition, five studies assessed NLR differences among mild, moderate, and severe asthma groups. The comparison between mild and moderate asthma showed a statistically significant difference (MD = −0.41; 95% CI: −0.64 to −0.18, *p* = 0.0005). The mean difference between mild and severe asthma was −3.10 (95% CI: −6.26 to 0.06; *p* = 0.05), suggesting a trend toward higher NLR in severe cases. The comparison between moderate and severe asthma revealed a non-significant difference (MD = −2.44; 95% CI: −5.31 to 0.44; *p* = 0.1), although the trend suggested higher NLR levels in severe cases (Figure 3B).

### Subgroup analyses by age and country development status

**Figure 4:**
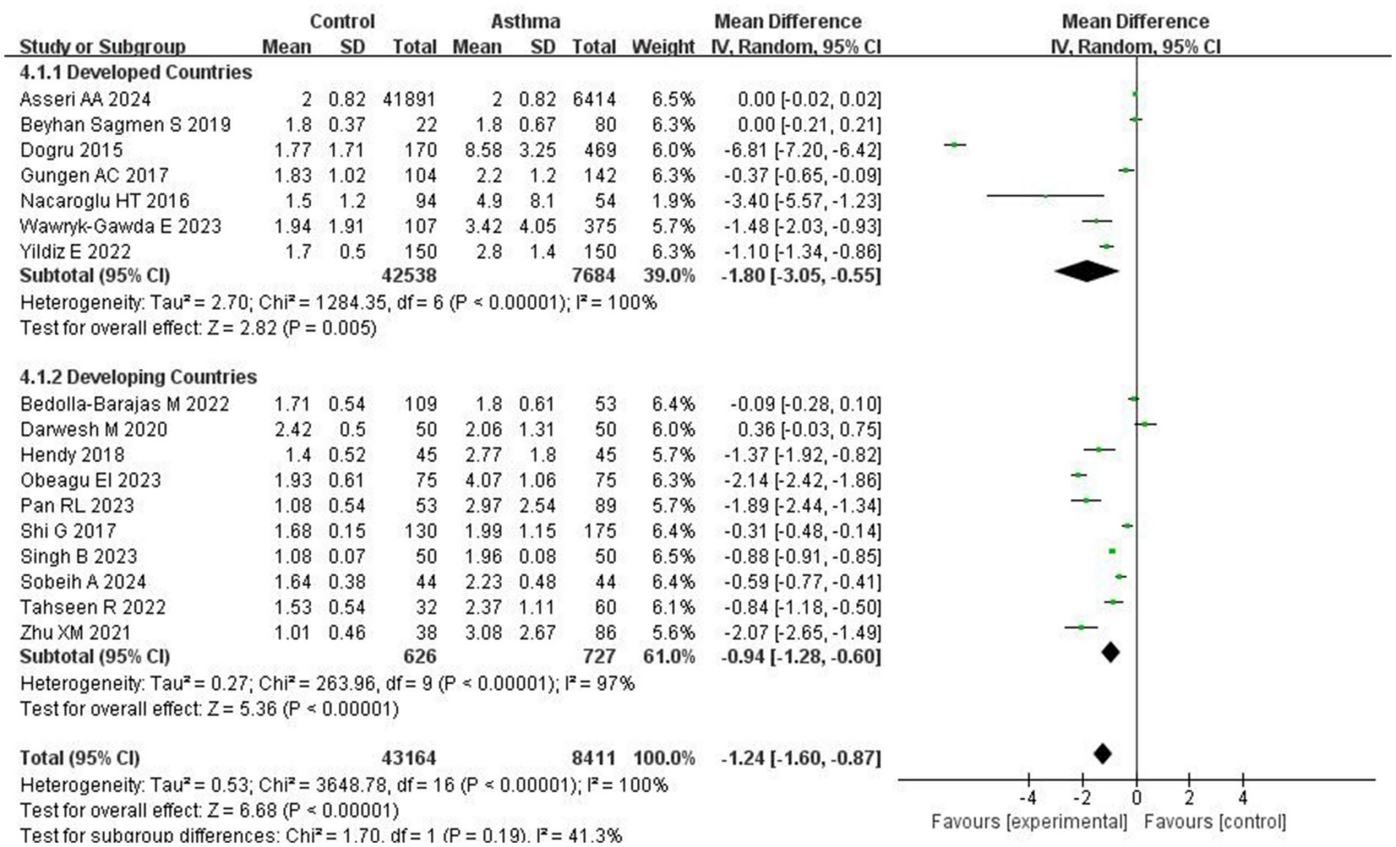
Forest plots of NLR across asthma patient subgroups by country development status.

**Figure 5:**
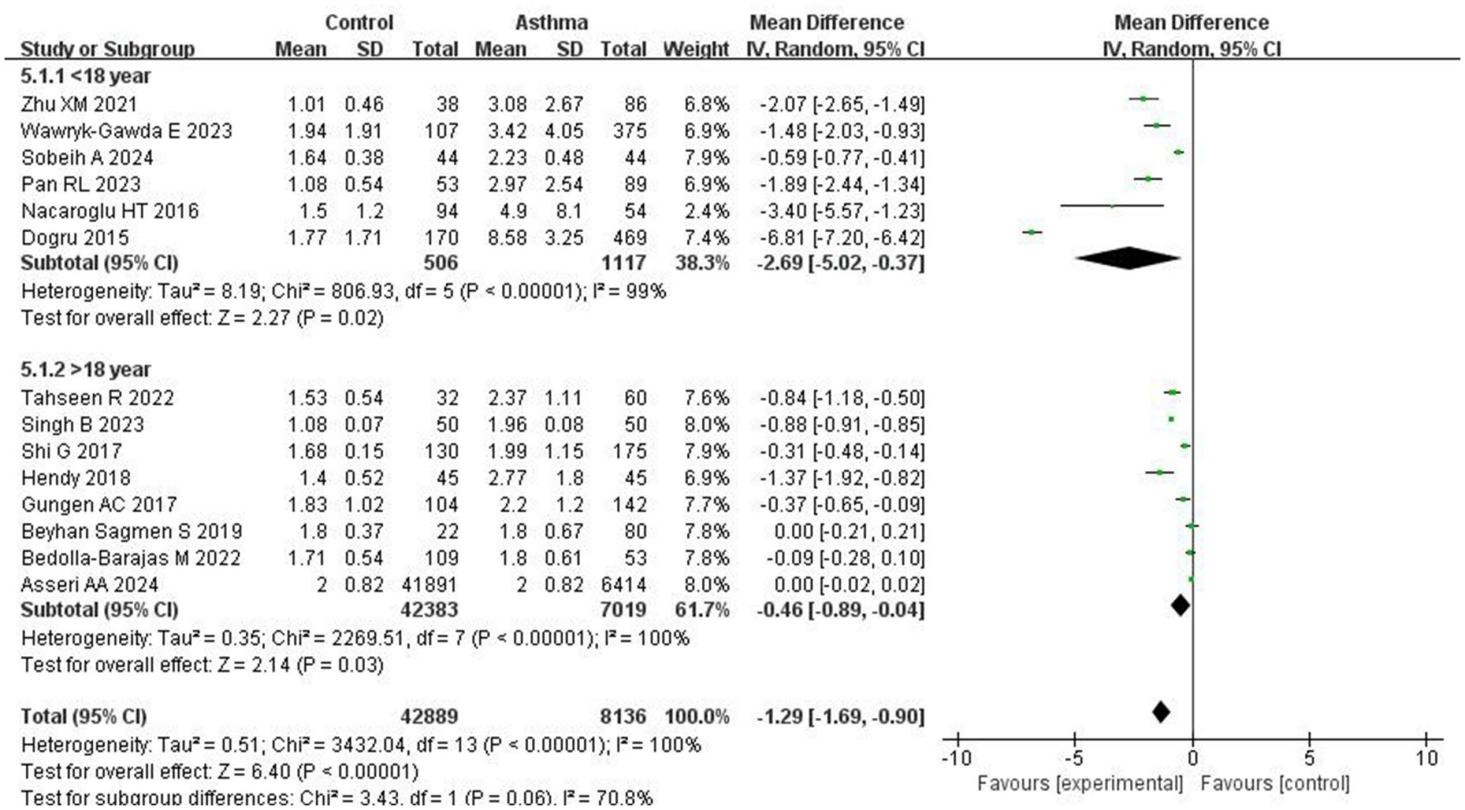
Forest plots of NLR across asthma patient subgroups by age.

Subgroup analyses were performed based on patient age and the development status of the countries. In studies from developed countries, the NLR was significantly higher in asthma patients compared to controls (MD = −1.80; 95% CI: −3.05 to −0.55, *p* = 0.005). Similarly, studies conducted in developing countries also showed a significant difference, with a pooled MD of −0.94 (95% CI: −1.28 to −0.60; *p* < 0.0001; I^2^ = 97%; Figure 4). Age-based subgroup analysis showed that in studies involving patients under 18 years, asthma patients had significantly higher NLR levels than controls (MD = −2.69; 95% CI: −5.02 to −0.37; *p* = 0.02; I^2^ = 99%). For those over 18 years of age, a significant difference was also observed (MD = −0.46; 95% CI: −0.89 to −0.04; *p* = 0.03; I^2^ = 100%; Figure 5).

### Sensitivity analysis

Sensitivity analyses were performed by sequentially excluding individual studies from the Control vs. Asthma and NSA vs. SA comparisons. The results remained consistent, indicating no single study significantly influenced the overall effect size. In the Control vs. Asthma group, all 17 studies showed significant negative mean differences (MDs), with I^2^ ranging from 96 to 100%. In the NSA vs. SA group, all studies similarly reported significant negative MDs, with I^2^ ranging from 90 to 96%, confirming the robustness of the meta-analysis findings (Table 4).

**Table 4:** Sensitivity analysis of the association between NLR and asthma.

Study	MD	95%CI	I^2^	*p*
Control vs. Asthma				
Nacaroglu	−1.08	−1.48,-0.69	100	<0.0001
Pan	−1.09	−1.49,-0.68	100	<0.0001
Dogru	−0.75	−1.09--0.40	100	<0.0001
Shi G	−1.19	−1.60,-0.78	100	<0.0001
Hendy	−1.12	−1.52,-0.71	100	<0.0001
Zhu	−1.08	−1.48,-0.67	100	<0.0001
Beyhan Sagmen	−1.21	−1.62,0.80	100	<0.0001
Asseri	−1.27	−2.02,-0.53	100	<0.0001
Wawryk-Gawda	−1.11	−1.52,-0.71	100	<0.0001
Darwesh	−1.23	−1.64,-0.82	100	<0.0001
Singh	−1.30	−1.85,-0.75	99	<0.0001
Yildiz	−1.13	−1.54,-0.73	100	<0.0001
Tahseen	−1.15	−1.56,-0.75	100	<0.0001
Gungen	−1.18	−1.59,-0.78	100	<0.0001
Sobeih	−1.17	−1.58,-0.76	100	<0.0001
Bedolla-Barajas	−1.21	−1.62,0.79	100	<0.0001
Obeagu	−1.06	−1.46,-0.67	100	<0.0001
NSA vs. SA				
Nacaroglu	−2.02	−2.84,-1.20	96	<0.0001
Pan	−2.11	−2.92,-1.30	96	<0.0001
Shi	−1.44	−2.12,-0.75	94	<0.0001
Hendy	−2.21	−3.07.-1.34	96	<0.0001
Beyhan	−2.66	−3.91,-1.41	96	<0.0001
Mochimaru	−2.69	−4.11,-1.26	96	<0.0001
Asseri	−1.13	−1.71,-0.55	90	<0.0001
Gungen	−2.49	−3.48,-1.50	96	<0.0001

### Assessment of publication bias

**Figure 6:**
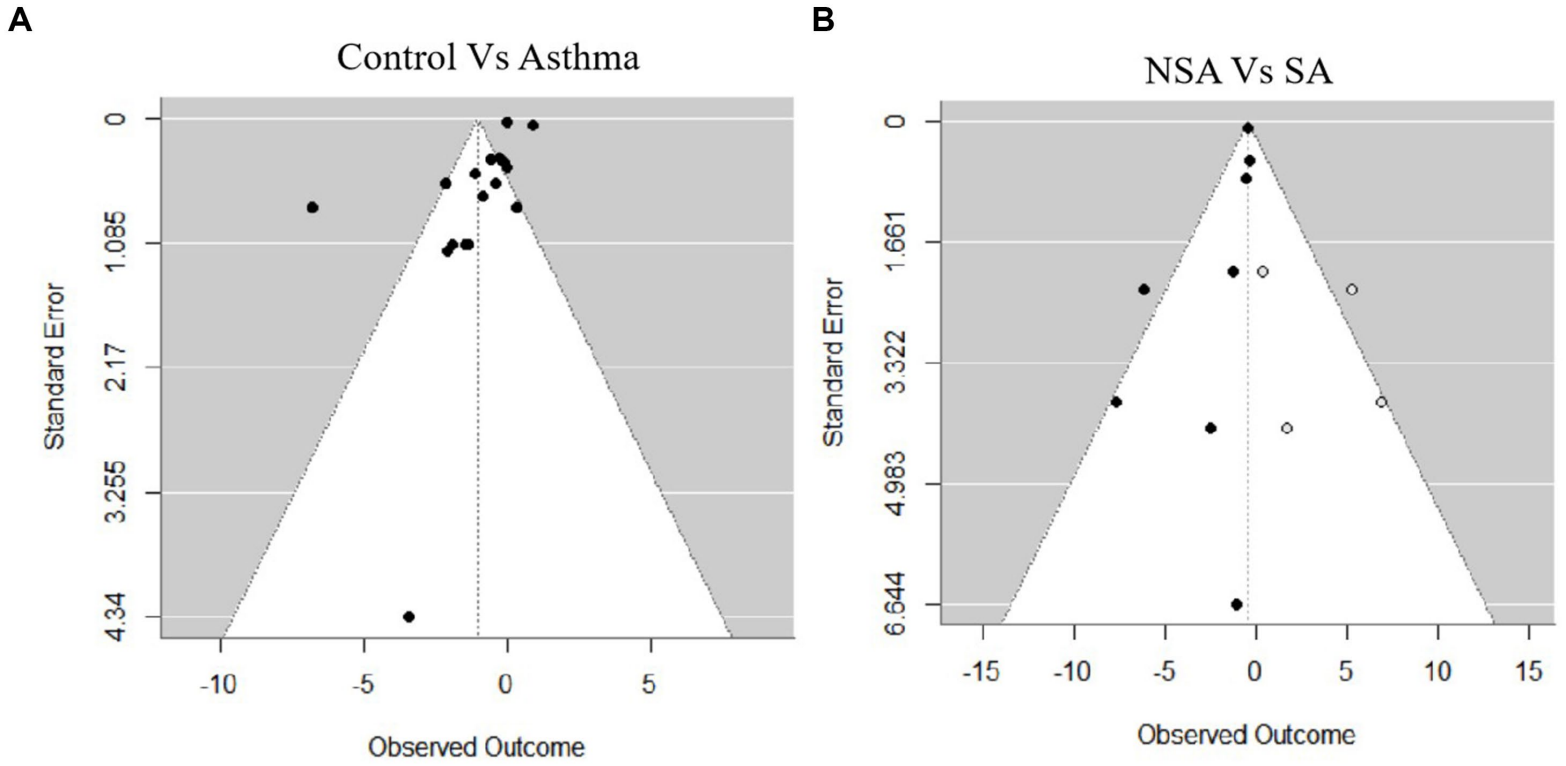
Funnel plots for publication bias. **(A)** Control vs. asthma. **(B)** Non-severe asthma (NSA) vs. severe asthma (SA).

Publication bias was assessed using both Egger’s regression test and Begg’s rank correlation test. In the comparison between the Control and Asthma groups, the Egger’s test indicated a trend toward funnel plot asymmetry (z = −1.9374, *p* = 0.0527), while Begg’s test showed no evidence of publication bias (Kendall’s tau = 0.0075, *p* = 0.9670; Figure 6A). For the comparison between NSA and SA, Egger’s test revealed potential publication bias (z = −2.0570, *p* = 0.0397). To explore the potential impact of this bias, a trim-and-fill analysis was conducted, which suggested that four missing studies were needed to restore symmetry. After adjustment, the pooled MD decreased to −0.41 (95% CI: −0.59 to −0.22, I^2^ = 0, *p* < 0.001). Begg’s test showed no evidence of bias (Kendall’s tau = −0.4286, *p* = 0.1789; Figure 6B).

### ROC analysis

**Figure 7:**
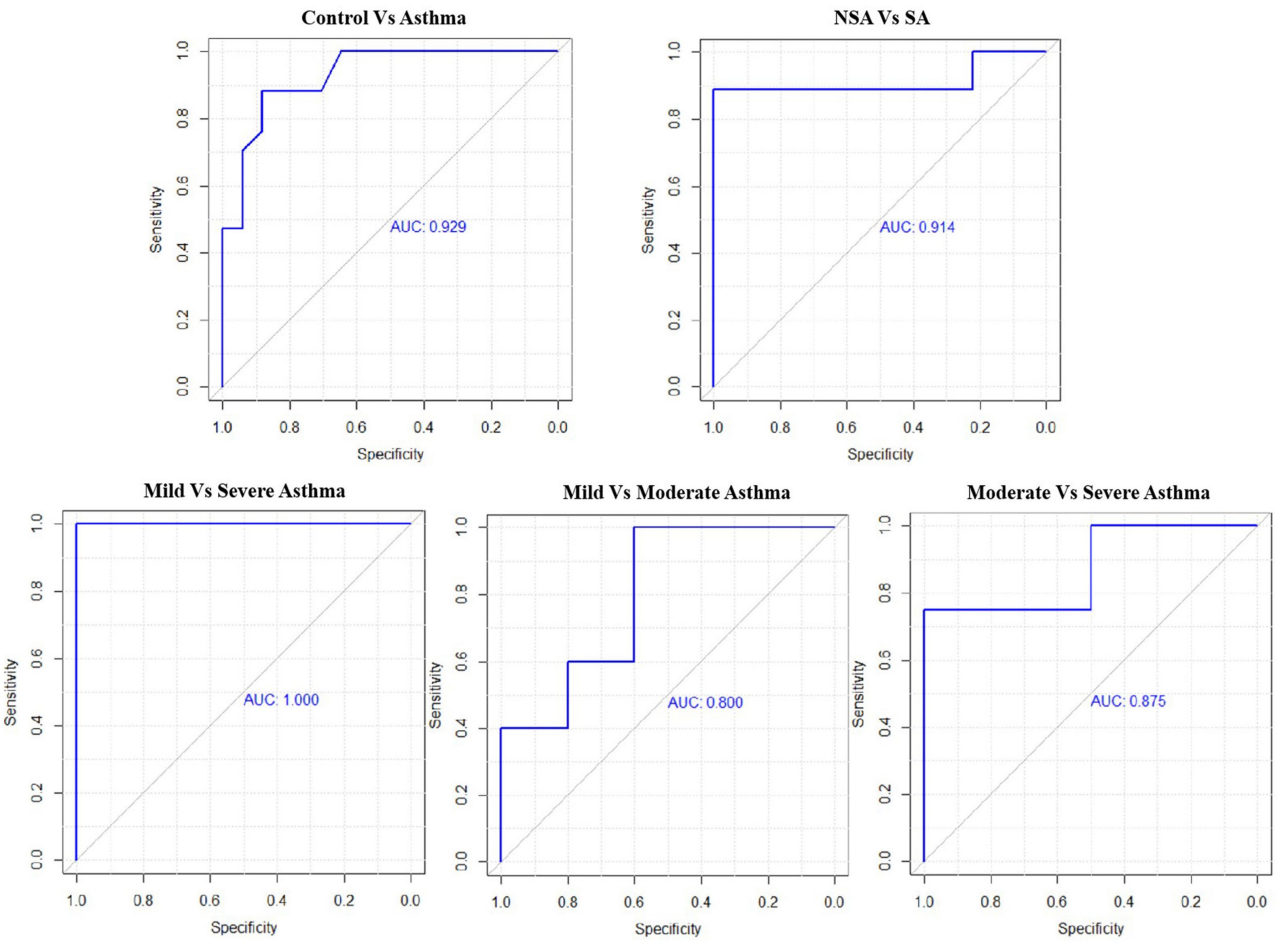
Receiver operating characteristic (ROC) curves for NLR across asthma severity subgroups.

ROC analysis showed that NLR had good diagnostic performance across various asthma group comparisons. For distinguishing Asthma from Control, the AUC was 0.929 with a cutoff of 1.95 (sensitivity: 88.2%, specificity: 88.2%). Between NSA and SA, the AUC was 0.914 (cutoff: 2.44; sensitivity: 88.9%, specificity: 100%). The comparison of Mild vs. Moderate Asthma yielded an AUC of 0.800 (cutoff: 2.12; sensitivity: 100%, specificity: 60%). For Mild vs. Severe Asthma, the AUC reached 1.000, with both sensitivity and specificity at 100% (cutoff: 2.21). In Moderate vs. Severe Asthma, the AUC was 0.875 (cutoff: 3.55; sensitivity: 75%, specificity: 100%; Figure 7; Table 5).

**Table 5:** Predict value of NLR among different exacerbated asthma groups.

Comparison	Cutoff	Sensitivity	Specificity	AUC
Control vs. Asthma	1.95	0.882	0.882	0.929
NSA vs. SA	2.44	0.889	1	0.914
Mild vs. Moderate	2.12	1	0.600	0.800
Mild vs. Severe	2.21	1	1	1
Moderate vs. Severe	3.55	0.750	1	0.875

## Discussion

This systematic review and meta-analysis evaluated the NLR as a biomarker reflecting systemic inflammation in asthma and its association with disease severity. Our findings demonstrate that NLR levels are significantly higher in asthma patients compared to healthy controls, with marked differences between severe and non-severe asthma groups. Notably, NLR effectively distinguished mild from moderate asthma, and showed a trend toward significance when comparing mild and severe asthma. However, the difference between moderate and severe asthma did not reach statistical significance.

These results align with previous meta-analyses by Huang et al (27) and Tahseen et al (28), who reported elevated NLR levels in asthma patients, particularly during exacerbations and in severe cases. Huang et al., however, included only six studies and focused primarily on acute exacerbations, while Tahseen et al. found significantly higher NLR levels in severe asthma compared to non-severe cases, suggesting a possible association between NLR and asthma severity. However, both studies were limited by small sample sizes and lacked detailed stratification across multiple severity levels.

The elevated NLR observed in asthma patients, particularly those with severe asthma, can be understood through the pathophysiology of disease. Asthma is characterized by chronic airway inflammation involving both neutrophils and lymphocytes (29, 30). Neutrophils, typically responsible for the innate immune response against infections, are often elevated in the airways of patients with asthma (31, 32). Their accumulation contributes to persistent inflammation, tissue injury, and airway remodeling—features that correlate with increased disease severity. Conversely, lymphocytes mediate allergic responses and adaptive immunity (33). An elevated NLR reflects a shift toward neutrophilic dominance and systemic inflammation, potentially serving as a surrogate marker for asthma severity and immune dysregulation. This pathophysiological relationship is supported by our ROC analysis, which demonstrated high diagnostic performance of NLR in distinguishing asthma patients from healthy controls (AUC = 0.929) and severe asthma from non-severe asthma (AUC = 0.914). However, the lower accuracy observed when differentiating mild from moderate asthma (AUC = 0.800) suggests that NLR may be less sensitive to subtle inflammatory changes in less advanced disease stages. This discrepancy may be attributed to overlapping inflammatory phenotypes in these stages (34), especially in patients with established airway remodeling or chronic neutrophilic burden. Additionally, relatively limited number of direct comparisons may have reduced the statistical power to detect subtle differences.

Subgroup analyses revealed significant geographic and age-related disparities in NLR levels Asthma patients in developed countries exhibited larger NLR differences compared to controls than those in developing regions, likely due to higher detection of severe phenotypes, greater environmental pollutant exposure (e.g., PM2.5), and improved healthcare access (35, 36). Conversely, underdiagnosis and limited treatment in developing areas may attenuate NLR elevations. Age stratification showed pediatric patients had markedly higher NLR differences than adults, potentially reflecting immature immune regulation favoring neutrophilic inflammation. Adults’ lower NLR differences may result from long-term anti-inflammatory therapy or adaptive immune modulation (16, 37). High heterogeneity across subgroups (I^2^ > 97%) underscores the influence of individual and environmental confounders. In addition to geographic and age-related factors, the variability in how asthma severity was defined across the included studies may also have contributed to the observed heterogeneity. Although all studies applied objective or guideline-based criteria, such as pulmonary function (FEV₁%), FeNO levels, bronchial provocation tests, reversibility testing, or adherence to GINA guidelines, there was no single unified standard across studies. Nonetheless, the consistent use of standardized clinical tools ensures a reasonable level of comparability and supports the validity of the pooled findings.

Several biomarkers have been integrated into clinical practice to guide treatment decisions in asthma, particularly in patients with severe or treatment-resistant disease. Among these, blood eosinophils, serum IgE, fractional exhaled nitric oxide (FeNO), and periostin are the most established and widely used, especially in identifying type 2 (T2)-high phenotypes that may benefit from corticosteroids or biologic therapies (38, 39). However, these biomarkers have limitations, including variable availability, higher cost, and limited utility in certain asthma phenotypes, such as those with non-eosinophilic or mixed inflammatory patterns. In this context, there is growing interest in identifying practical, affordable, and broadly applicable biomarkers to support individualized asthma management.

The NLR, derived from routine blood tests, has been proposed as an accessible marker of systemic inflammation. Although not specific to asthma, our meta-analysis demonstrates a consistent association between elevated NLR and greater disease severity. While this study did not stratify patients by inflammatory endotype, these findings support the potential utility of NLR as a supplementary tool, particularly in settings where conventional biomarkers are unavailable or less informative.

Despite these efforts, several limitations should be acknowledged. First, the overall heterogeneity observed in the pooled analyses was substantial, which may affect the generalizability of the findings. While we employed a random-effects model and conducted subgroup and sensitivity analyses to explore potential sources, residual variability likely remains. Second, although NLR levels increased with asthma severity, its ability to clearly differentiate between moderate and severe asthma stages was limited, suggesting that NLR alone may not fully capture nuanced inflammatory changes across disease progression. Third, most of the included studies were retrospective in design, introducing potential selection and information biases and underscoring the need for future prospective studies to validate and refine the clinical applicability of NLR in asthma management.

## Conclusion

This systematic review and meta-analysis confirms that the NLR is elevated in asthma patients compared to healthy controls, with levels increasing alongside disease severity. Subgroup analyses revealed more pronounced NLR differences in pediatric populations and studies from developed countries. While NLR effectively distinguished asthma and severe cases, its ability to differentiate between moderate and severe asthma was limited. These findings support the potential role of NLR as a biomarker for asthma identification and severity stratification. Future prospective studies are needed to validate standardized thresholds and to explore its combined use with other indicators for improved precision across all severity stages.

## Data Availability

The original contributions presented in the study are included in the article/supplementary material, further inquiries can be directed to the corresponding author/s.
